# Microwave-Assisted Extraction of Phenolic Antioxidants from Potato Peels

**DOI:** 10.3390/molecules16032218

**Published:** 2011-03-07

**Authors:** Ashutosh Singh, Kebba Sabally, Stan Kubow, Danielle J. Donnelly, Yvan Gariepy, Valérie Orsat, G.S.V. Raghavan

**Affiliations:** 1 Department of Bioresource Engineering, McGill University, 21,111 Lakeshore Rd., Sainte-Anne-de-Bellevue, QC H9X 3V9, Canada; 2 School of Dietetics & Human Nutrition, McGill University, QC H9X 3V9, Canada; 3 Department of Plant Science, McGill University, QC H9X 3V9, Canada

**Keywords:** response surface method, chlorogenic acid, ferulic acid, ascorbic acid, DPPH, potato peel

## Abstract

A response surface method was used to optimize the microwave-assisted extraction parameters such as extraction time (*t*) (min), solvent (methanol) concentration (*S*) (v/v) and microwave power level (*MP*) for extraction of antioxidants from potato peels. Max. total phenolics content of 3.94 mg g^−1^ dry weight (dw) was obtained at *S* of 67.33%, *t* of 15 min and a *MP* of 14.67%. For ascorbic acid (1.44 mg g^−1^ dw), caffeic acid (1.33 mg g^−1^ dw), ferulic acid (0.50 mg g^−1^ dw) max contents were obtained at *S* of 100%, *t* of 15 min, and *MP* of 10%, while the max chlorogenic acid content (1.35 mg g^−1^ dw) was obtained at *S* of 100%, *t* of 5 min, and *MP* of 10%. The radical scavenging activity of the extract was evaluated by using the DPPH assay and optimum antioxidant activity was obtained at *S* of 100%, *t* of 5 min, and *MP* of 10%.

## Abbreviations

*Phen*_tot_: total phenolic content
*AA*: ascorbic acid
*ChloA*: chlorogenic acid
*CafA*: caffeic acid
*FerA*: ferulic acid
DPPH: 1,1-diphenyl-2-picrylhydrazyl
MAE: microwave-assisted extraction
RSM: response surface methodology
R^2^: Correlation coefficient

## 1. Introduction

The potato (*Solanum tuberosum* L.) processing industry is one of the largest food processing industries in Canada and the United States. In 2008, some 21.8 million tonnes of potatoes were produced, and over 50% of were processed, mostly for French fries (60%) and chips (22%). Waste generation from potato processing ranges from an estimated 33% to 35% of the original potato fresh weight [1]. At present, a large proportion of this waste is used as an animal feed and for the production of bio-fuels. Recent studies, however, have also described the use of potato peels as a source of natural antioxidants to increase the shelf life of several food products, including soybean oil [2] and processed lamb meat [3]. 

Potatoes are a major dietary source of phenolics and a number of antioxidants [4,5,6,7]. These antioxidants obtained from potato have free radical scavenging effects, and decrease the risk of coronary heart diseases [8,9] by reducing cholesterol accumulation in the blood serum and by enhancing the resistance of vascular walls [10]. From a dietary point of view, potatoes are second only to tomatoes (*Solanum lycopersicum* L*.*) in the total intake of polyphenols by humans [10]. Potato tuber skin contains more polyphenols than the cortex and pith [11,12]. The recovery of these valuable phenolics from the potato peel waste could improve the economics of the potato processing industries and also provide a cheaper and effective alternative to synthetic antioxidants which are commercially utilized in the food industies [13,14,15]. 

Conventionally, antioxidants from potato peels are extracted using traditional methods such as Soxhlet and heat reflux [16]. These methods have been associated with longer extraction times, high solvent consumption and increased risk of degradation of heat-labile constituents. Dai *et al*. [17,18] recently evaluated a novel microwave-assisted extraction (MAE) process which used microwave energy to heat the solvent and the sample in order to extract a specific compound from the sample into the solvent. MAE offers many advantages such as shorter extraction times, lower solvent consumption, and higher selectivity towards target molecules [19,20]. MAE has been used to extract bioactive compounds from a wide variety of plants and natural residues [21]: secoisolariciresinol diglucoside from flaxseeds (*Linum usitatissimum* L.) [22,23], resveratrol and phenolic antioxidants from peanut (*Arachis hypogæa* L.) skin [24,25], flavonoids from Chinese herbs [20], solanesol from potato leaves and stems [26], pigments from paprika (*Capsicum annuum* L*.*) [27], and pectin from apple (*Malus domestica*) pomace [19].

Current consumer interest in natural food additives and the beneficial effects of potato antioxidants directed the current study towards developing an MAE process for the extraction of antioxidants from potato peel. A response surface method (RSM) was used to study the effects of solvent concentration, extraction time and microwave power level on the concentration of phenolics in the extract.

## 2. Results and Discussion

### 2.1. Optimization of conditions for phenolic extraction

Results from the preliminary investigation of MAE conditions indicated that temperature measured inside the vessel, which determines the efficiency of the extraction process, depends on the volume of the solvent added, microwave irradiation time and power level applied. It was observed that solvent concentration (*S*) and time (*t*) significantly influenced total phenolics content (*p* ≤ 0.0001 and *p* ≤ 0.005, respectively) as did their quadratic terms (*S*^2^ and *t*^2^; *p* ≤ 0.0001 and *p* ≤ 0.05, respectively) (Table 1), whereas neither microwave power (*MP)* nor itsquadratic term (*MP*^2^), nor any parameter interactions (e.g., *S* × *t*, *MP* × *S*...) had any effect (*p* > 0.05). 

**Table 1:** ANOVA for the effect of solvent concentration (*S*), and time (*t*) on total phenolics.

Source	SS	DF	MS	F-Value	*p-value*
**Model**	2.83	4	0.71	56.34	≤0.0001
***S*-Solvent Concentration**	0.45	1	0.45	35.90	≤0.0001
***t*-Time**	0.18	1	0.18	14.37	0.0026
***MP*- Power**	1.009 × 10^−3^	1	1.009 × 10^−3^	0.21	0.6636
***S*×*t***	5.065 × 10^−3^	1	5.065 × 10^−3^	1.04	0.3428
***S*×*MP***	3.176 × 10^−7^	1	3.176 × 10^−7^	6.942 × 10^−5^	0.9938
***T*×*MP***	1.788 × 10^−4^	1	1.788 × 10^−4^	0.037	0.8538
***S*^2^**	2	1	2	159.64	≤0.0001
***t*^2^**	0.078	1	0.078	6.20	0.0284
***P^2^***	0.020	1	0.020	4.11	0.0821
**Residual**	0.15	12	0.013		
**Lack of Fit**	0.13	8	0.016	2.69	0.1773
***R*^2^**	0.95				

The predicted regression model for total phenolics (*Phen*_tot_) can be described in terms of coded factors, with *S* and *t* as the significant factors:

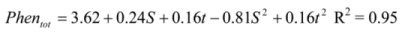
(1)


**Figure 1:**
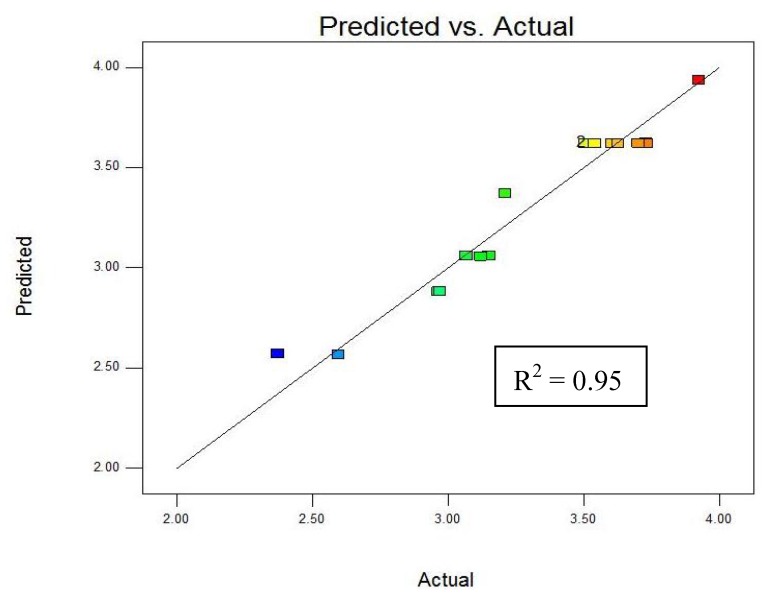
Predicted (mg g^−1^) *vs.* measured (mg g^−1^) total phenolics.

A plot of measured *vs.* predicted values of *Phen*_tot_ (Figure 1) shows the close agreement between these values, suggesting that the model and resulting response surface can be used to predict total phenolics under different experimental conditions. 

The results obtained were in accordance with those of Rodriguez de Sotillo *et al.* [16], who studied extraction and stability of phenolics extracted from waste potato peel using aqueous solutions of methanol, and observed that with an increase in temperature, the total phenolic yield increased significantly, however it also changed the composition of the phenolics obtained. Similar results were obtained in the present study where the total phenolic yield and composition of the phenolics varied with changes in experimental parameters. 

Similar regression models were developed for ascorbic acid and other individual phenolics using the software. Only the significant factors were used to obtain the predicted concentration for the individual phenolics. For ascorbic acid (*AA*) only *MP* showed a significant and inverse effect on ascorbic acid content (*p* ≤ 0.01), while all other parameters, interactions and quadratic terms were not significant (*p* > 0.05). Equation 2 shows the relationship between *MP* and *AA* content:

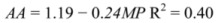
(2)


**Figure 2:**
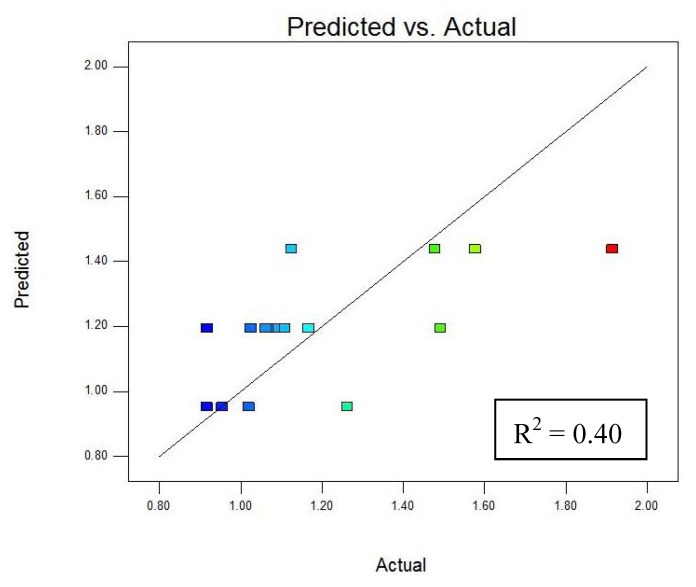
Predicted *vs*. actual ascorbic acid content (mg g^−1^).

This result indicates that changes in solvent concentration and extraction time had no significant effect on the concentration of the *AA* obtained, but power had considerable effect on the response when MAE was used to extract *AA* from freeze dried potato peel. A plot of predicted *vs*. actual *AA* content (Figure 2) shows that experimental values are widely scattered, likely because *AA* is heat sensitive and the temperature reached as a result of the microwave power levels and solvent concentrations used was high enough to oxidize it.

For individual phenolics like chlorogenic acid (*ChloA*) and caffeic acid (*CafA*) only *S* showed a significant and positive effect (*p* ≤ 0.05), while all other parameters, interactions and quadratic terms were not significant (*p* > 0.05). Equations 3 & 4 show the relationship between *S* and *ChloA* and *CafA* content:

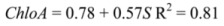
(3)

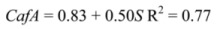
(4)


For ferulic acid (*FerA)* both *S* and *t* showed a significant and positive effect on its content (*p* ≤ 0.05), while all other parameters, interactions and quadratic terms were not significant (*p* > 0.05). Equation 5 shows the relationship between *S, t* and *FerA* content:

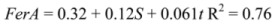
(5)


### 2.2. Radical scavenging activity

The well known DPPH assay was used to measure the radical scavenging ability of potato peel extracts. Only solvent concentration had a highly significant effect (*p* ≤ 0.05) on the DPPH test value, while all other parameters, interactions and quadratic terms were not significant (*p* > 0.05). Equation 6 shows the relationship between *S* and radical scavenging ability as measured by the DPPH test:

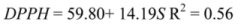
(6)


### 2.3. Response surface analysis

Regression models were used to predict the effect of the three independent variables on *Phen*_tot_, *AA*, *ChloA*, *CafA*, *FerA* content and DPPH radical scavenging activity. The relationships between independent and dependent variables were illustrated in three-dimensional response surfaces.

**Figure 3:**
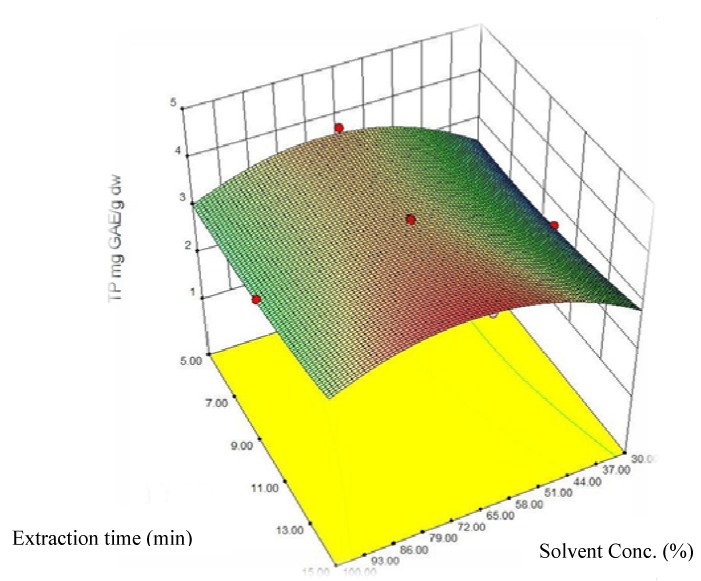
Response surface plot of the effect of methanol (MeOH) concentration (% v/v) and extraction time (min) on total phenolics (TP) content of potato peel extract.

The response surface of the effect of independent variables time and solvent concentration on total phenolics (Figure 3) shows that at constant power level of 20%, total phenolic content of the extract increased with increase in extraction time.

The linear and squared terms of the solvent concentration had a significant impact on the model. Similar results were obtained when extracting phenolics from peanut skin [24]. In this study, *Phen*_tot_ content increased with an increase in methanol concentration up to 65–70% and then declined. The optimum extraction condition for total phenolics was estimated to be 67.33% aqueous methanol, 15 minutes extraction time, and 14.67% power. The maximum predicted *Phen*_tot_ content under such conditions would be 3.94 ± 0.21 mg GAE g^−1^. Microwave power had no significant impact on the *Phen*_tot_, content though at higher power levels *Phen*_tot_ increased to predicted value by reducing the extraction time and increasing solvent concentration. The *Phen*_tot_ value obtained under the experimental conditions used ranged from 1.2 ± 0.8 to 3.9 ± 0.21 mg GAE g^−1^ dry potato peel powder. Several researchers using conventional method of extraction reported a range of 1.51 ± 0.17 to 3.32 ± 0.12 mg GAE g^-1^ [11], 0.48 mg g^-1^ [16]. Al-Weshahy *et al.* [11] reported that the highest *Phen*_tot_ values were found in coloured potato varieties. Other researchers have also shown that coloured potato varieties have more GAE total phenolic content [4,28]. In our study we used the brown skin variety ‘Russett Burbank’ for MAE optimization. Our results indicate that MAE exhibited a better efficiency than conventional methods of extraction (Table 2).

**Table 2:** Comparison of result obtained in the present study with those reported in the literature.

Component	Present study	Reported literature	Reference and study group
Quantified			
***Phen*_tot_**	3.94 ± 0.21 mg /g dw	1.51 ± 0.17 − 3.31 ± 0.12 mg/g dw	[11]
***AA***	1.44 ± 0.5 mg /g dw	-	-
***ChloA***	1.35 ± 0.18 mg /g dw	0.78 ± 0.01 − 2.79 ± 0.12 mg/g dw	[11]
***CafA***	1.33 ± 0.06 mg /g dw	0.26 ± 0.01 − 0.72 ± 0.29 mg/g dw	[11]
***FerA***	0.5 ± 0.02 mg /g dw	0.6 ± 0.1 – 3.9 ± 0.4 mg/100 g dw	[29]
**DPPH**	74 ± 5.5%	-	-

**Figure 4:**
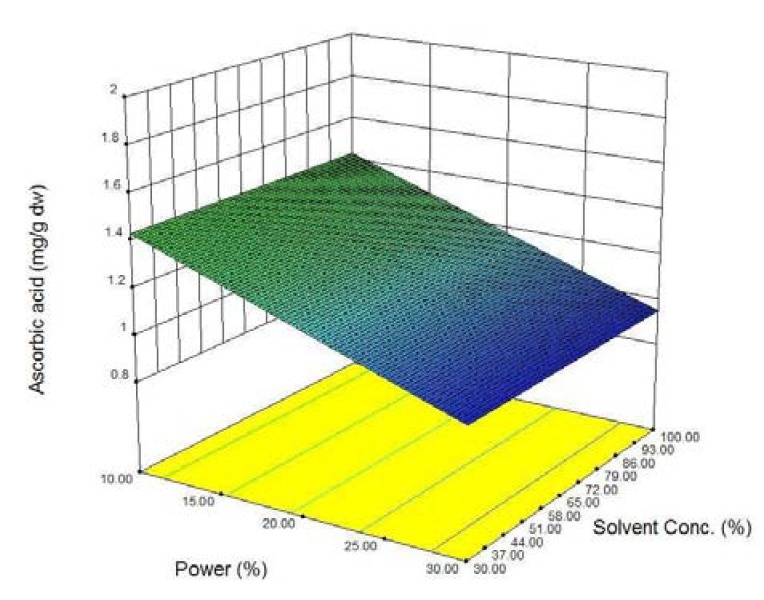
Response surface plot of the effect of methanol (MeOH) concentration and effect of microwave power on ascorbic acid content of Potato peel extract.

The response surface plot for *AA* content (Figure 4) shows that solvent concentration was highly significant, whereas time and microwave power may play only minor role in the extraction process within the ranges tested. 

Microwave power affects the temperature, *i.e.*, an increase in power level will increase the temperature of the extraction process. *AA* heat-sensitivity is reflected in a significant decrease in its concentration in the extracts achieved with increasing power. Extending the extraction time resulted in a linear increase in the *AA*. Increasing the solvent concentration increased the *AA* content in the extract. Further investigation with lower power levels and use of modified MAE techniques such as low-temperature vacuum MAE and nitrogen-protected MAE are needed to maximize the yield of *AA* from potato peel samples [30]. 

Similar response plots (not shown) were obtained for the individual phenolic compounds measured using HPLC. Optimized experimental conditions derived from the plots, and the resultant predicted individual phenolic compound levels are given in Table 3. 

**Table 3:** Optimal conditions and predicted contents of ascorbic, chlorogenic, caffeic acid, ferulic acid and total phenolic content of the extract.

Predicted responses	Methanol concentration (%v/v)	Extraction time (min)	Power level (W)	Predicted content mg g^−1^/ dw
***AA***	100	15	10	1.44 ± 0.5
***ChloA***	100	5	10	1.35 ± 0.18
***CafA***	100	15	10	1.33 ± 0.06
***FerA***	100	15	10	0.5 ± 0.02
***Phen*_tot_**	67.33	15	14.67	3.94 ± 0.21
**DPPH**	100	5	10	74 ± 5.5%

For *ChloA* and *CafA* the solvent concentration played a significant role, with their concentration rising with an increase in solvent concentration. The linear relationship of factors such as extraction time and power on the content of an individual phenolic compound might be attributable to the selection of a limited range for these treatment parameters in this study. Total phenolic yield increased with increase in power level, but the composition of the individual phenolic compounds varied significantly. The values obtained for individual phenolics like *ChloA* and *CafA* were in accordance with the literature and were significantly higher than those obtained by conventional extraction methods (e.g., [4,11,31,32,33]). 

The DPPH method was used to measure the free radical scavenging activity of the potato peel extract. The assay is a simple, rapid and convenient method, independent of sample polarity. These advantages make DPPH an ideal method that can be used to model the reaction between antioxidants and lipids in food system [34,35]. The optimum extraction condition that provided maximum free radical inhibition (74%) were predicted to be 100% (v/v) methanol concentration, power level of 10% and extraction time of 5 minutes. Experimental results suggested that the inhibition increased with increase in concentration up to 65% (v/v) and then remained constant up to 100% (v/v). 

To the best of our knowledge, there have been no previous reports addressing the optimization of the MAE process for potato peel. We were able to optimize the phenolic content in the MAE extract while considering the efficiency, economy and feasibility of the process. Predictions achieved through the regressions developed indicated that different combinations of independent variables are required to maximize each dependent variable in the extract. For individual phenolics, the solvent concentration plays a major role because different aqueous methanol mixes have different dielectric properties [36,37,38]. This change in the dielectric property with varying methanol fractions has a profound effect on the extraction of phenolics from potato peel. 

## 3. Experimental

### 3.1. Materials and reagents

Potatoes (cv. ‘Russet Burbank,’) were obtained from the Elite Potato Centre at Bon Accord (NB, Canada). Tubers were washed and peeled with a mechanical peeler to obtain uniformity in thickness of the peel. The peel samples were lyophilized in a laboratory freeze-dryer (Thermo Savant Modulyod-115, NY, USA) until a constant weight was obtained. After drying, the samples were ground to pass a standard 150 µm sieve, thus ensuring uniformity and symmetry of particle size. The freeze-dried powder was kept in closed opaque containers at −20 °C until analysis. All reagents and solvents used were of HPLC grade (Fisher Scientific, Ottawa, ON, Canada)

### 3.2. Equipment and apparatus

MAE extraction was carried out with a focused open-vessel microwave system (Star System 2, CEM Matthews, USA) operating at 800 W maximum power and a frequency of 2.45 GHz. Power level used for the experiments were expressed as a percent of the power supplied within the microwave cavity. The maximum microwave output power in the cavity was calibrated using the protocol developed by Cheng *et al*., [39]. The regression equation (Equation 7) obtained from the calibration was used to convert % power level into Watts:

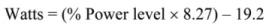
(7)


The mode of microwave power applied was intermittent with power on for 30 s min^−1^. 

### 3.3. Preparation of potato peel extracts

The phenolic compounds were extracted from freeze dried potato peels using 30, 65 or 100% (v/v) methanol (MeOH). Solvent (40 mL) was added to dried potato peel (2 g) in a Pyrex vessel and placed inside the microwave, where phenolics were extracted at different power levels (% W) over different periods of time (min). The extract was allowed to cool at room temperature and was then centrifuged at 10,000 rpm for 15 min. The supernatant was collected and used for total phenolics determination, free radical scavenging activity assay and HPLC analysis of the different phenolic compounds.

### 3.4. Determination of total phenolic compounds

Total soluble phenolics were determined using Folin-Ciocalteu reagent [40,41]. Potato peel extract solution (1 mL) was mixed with double distilled water (7.5 mL) and Folin-Ciocalteu reagent (0.5 mL) followed by 5% Na_2_CO_3_ solution (1 mL). The mixture was incubated at room temperature for 90 min and its absorbance was measured at 765 nm using a spectrophotometer (Ultrospec 2100pro, Biochrom Ltd., Cambridge, UK). A standard curve was plotted using different concentrations of gallic acid and the amount of total phenolic content was expressed in terms of gallic acid equivalents (GAE) in mg per 100 g of potato peel (dry weight).

### 3.5. Scavenging activity on 1,1-diphenyl-2-picrylhydrazyl (DPPH) radicals

The free radical scavenging activity of potato peel extract on DPPH radicals was measured according to the method proposed by Nair *et al*. [42] with some modifications. An aliquot (50 µL) of methanolic extract of potato peel was added to DPPH (1.5 mL, 3.94 mg/100 mL methanol). Free electrons present in DPPH get paired off in presence of antioxidants and the absorption decreases as the result of extinction of DPPH’s purple colour. Decolourisation was determined by measuring the absorption at 517 nm with the Ultrospec 2100pro spectrophotometer after 20 min. Experiments were conducted in triplicate and scavenging activity on DPPH radicals was expressed as the percentage (%) inhibition using following equation:

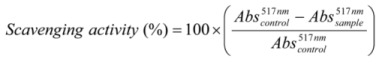
(8)


### 3.6. HPLC analysis

**Figure 5:**
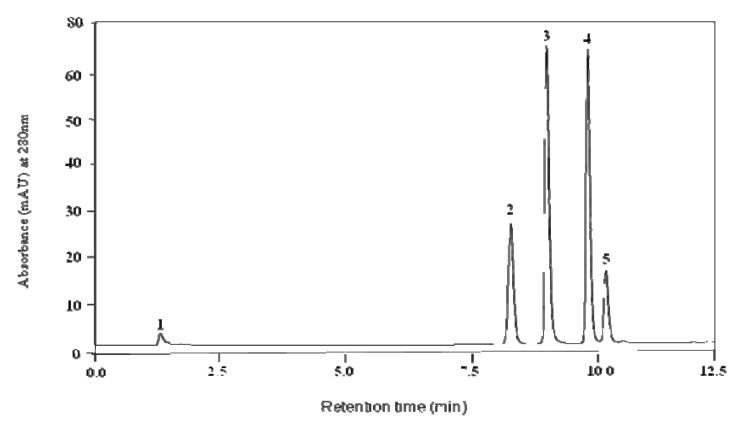
Chromatogram of standards: ascorbic acid (peak 1), chlorogenic acid (peak 2), caffeic acid (peak 3), ferulic acid (peak 4) and rutin (peak 5).

**Figure 6:**
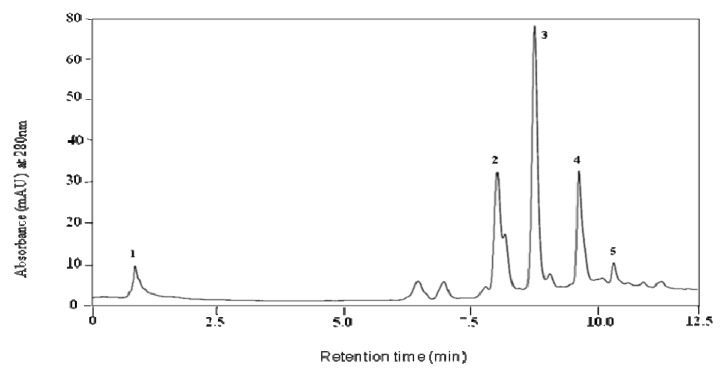
HPLC Chromatogram of a potato peel polyphenolic extract. Ascorbic acid (peak 1), chlorogenic acid (peak 2), caffeic acid (peak 3), ferulic acid (peak 4) and rutin (peak 5).

A Varian High Performance Liquid Chromatography (HPLC) system equipped with a tertiary pump, refrigerated auto-sampler and a UV/visible wavelength detector was used for sample analysis. Phenolic materials were separated using a reverse phase HPLC Gemini-NX (5 μm, 100 mm × 4.6 mm) column (Phenomenex, Inc., Torrance, CA, USA) equipped with a 4.6 mm × 2.0 mm guard column. The mobile phase was composed of solvent buffer A (10 mM formic acid, pH 3.5, with NH_4_OH) and buffer B (100% methanol with 5 mM ammonium formate). The solvent gradient was as follows: 0–1 min 100% buffer A, 1–5 min 0–30% buffer B, 5–6.5 min 30–70% buffer B, 6.5–8.5 min 70–100% buffer B. UV detection was conducted at 280 nm. A flow rate of 1.0 mL min^−1^ was used and 20 μL of sample were injected. Samples were analyzed in duplicates. Ascorbic acid, chlorogenic acid, caffeic acid and ferulic acid were analyzed by HPLC (Figure 5 and Figure 6).

### 3.7. Optimization of conditions for microwave-assisted extraction of phenolics

The conditions for microwave-assisted extraction were optimized with respect to effect of three independent variables: methanol percentage in methanol-water solvent mixture (*S*), microwave-power level (*P*), and extraction time (*t*) using a central composite design. The experimental design was developed using Design Expert (ver. 8, Stat-Ease, Inc., Minneapolis, MN, USA) to determine the effect of process parameters (variables), each at three equidistant levels(−1,0,+1) (Table 4) and their interaction on the response variables, such as total phenolic content (*Phen*_tot_), ascorbic acid content (*AA*), chlorogenic acid content (*ChloA*), caffeic acid content (*CafA*),, ferulic acid content (*FerA*) and scavenging activity on DPPH free radical (*DPPH*). The complete experimental design consists of 20 different combinations of factors including six replications of the centre points (Table 5), and all the experiments were replicated thrice to improve the analysis. The three levels of factors were selected based on preliminary experiments.

**Table 4:** Coded (individually for each treatment factor) and corresponding actual values of the independent variables analyzed in RSM.

Code	Solvent concentration (%v/v)	Time (min)	Power (%)
**-1**	30	5	10
**0**	65	10	20
**1**	100	15	30

Note: Power 10% = 63W; Power 20% = 146W and Power 30% = 229W.

### 3.8. Statistical analysis

All the statistical analyses were carried out using the Design Expert software**.** The fitness of the model was determined by evaluating the Fisher test value (*F*-Value), and the coefficient of determination (*R^2^*) as obtained from an analysis of variance (ANOVA) at 95% confidence (*p* ≤ 0.05) level.

**Table 5:** Central composite rotatable design for Response Surface Analysis of antioxidant extraction from potato peel using methanol.

Run	Methanol concentration (%v/v)	Time (min)	Power (%)	Total Phenolic (mg/g dw)	Ascorbic acid content (mg/g dw)	Chlorogenic acid content (mg/g dw)	Caffeic acid content (mg/g dw)	Ferulic acid content (mg/g dw)	DPPH (%)
1	0	0	0	3.626	1.109	0.62	0.63	0.34	57.27
2	0	0	-1	3.504	1.125	0.465	0.581	0.37	55.20
3	0	0	0	3.733	0.917	0.571	0.627	0.288	56.65
4	0	0	0	3.605	1.062	0.725	0.961	0.367	55.5
5	-1	0	0	2.595	1.025	0.258	0.284	0.162	34.03
6	0	0	0	3.701	1.492	0.658	0.742	0.319	57.55
7	-1	1	-1	2.964	1.577	0.362	0.642	0.238	42.35
8	0	-1	0	3.729	1.087	0.532	0.59	0.23	68.55
9	1	-1	-1	3.151	1.478	1.674	1.524	0.31	70.66
10	-1	-1	-1	2.051	0.92	0.328	0.228	0.16	52.33
11	1	1	1	3.001	1.012	0.774	0.892	0.374	70.46
12	0	0	0	3.515	1.097	0.435	0.493	0.342	56.67
13	1	1	-1	3.21	1.915	1.524	1.411	0.381	73.32
14	0	1	0	3.924	1.167	0.861	0.92	0.44	72.94
15	-1	-1	1	2.37	0.917	0.37	0.288	0.16	41.4
16	-1	1	1	2.969	1.02	0.279	0.407	0.206	48.66
17	1	0	0	3.12	1.106	1.033	1.19	0.396	66.45
18	1	-1	1	3.067	1.262	1.392	1.228	0.419	69.5
19	0	0	0	3.675	1.51	0.521	0.491	0.427	72.34
20	0	0	1	3.541	0.954	0.585	0.57	0.317	86.23

The central composite rotatable design uses least-squares regression to fit the experimental data to a quadratic model [24,43]. The quadratic model describes the relationship between response (*Y*) and the process parameters (*X_i_,X_j_,X_k_….*)is as follows:

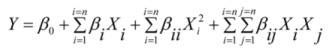
(9)
where *β*_0_ is the constant coefficient_,_*β*_i_ is the linear coefficient, *β*_ii_ is the quadratic coefficient for main process parameters and *β*_ij_ is the second order interaction coefficient of variables *i* and *j*, respectively. The 3D response surface graphs for the predicted value were plotted using the software’s tools.

## 4. Conclusions

Utilization of potato peel for extraction of beneficial phytonutrients such as phenolic antioxidants not only provides health benefits, but also adds value to the waste generated by the potato processing industries. In our study RSM proved to be effective in estimating the effect of three independent variables on the extraction of ascorbic acid and selected phenolics. Methanol concentration and extraction time played significant roles in extraction of individual phenolics. We were able to extract higher levels of phenolics from dried potato peel than the values reported number of previous studies, while using less solvent and considerably reducing the extraction time. Future work could focus on optimization and large scale extraction of selected phytonutrients from waste potato peels and their use as food additives. 
